# Historical and future carbon stocks in forests of northern Ontario, Canada

**DOI:** 10.1186/s13021-021-00184-5

**Published:** 2021-07-15

**Authors:** Michael T. Ter-Mikaelian, Alemu Gonsamo, Jing M. Chen, Gang Mo, Jiaxin Chen

**Affiliations:** 1 Ontario Forest Research Institute, Ontario Ministry of Natural Resources and Forestry, 1235 Queen Street E., Sault Ste. Marie, ON P6A 2E5 Canada; 2 School of Earth, Environment & Society, McMaster University, 1280 Main Street West, Hamilton, ON L8S 4S4 Canada; 3 Department of Geography and Planning, University of Toronto, 100 St. George St, Toronto, ON M5S 3G3 Canada

**Keywords:** Carbon balance, Boreal, Climate change, Shared socioeconomic pathways, Far North of Ontario

## Abstract

**Background:**

Forests in the Far North of Ontario (FNO), Canada, are likely the least studied in North America, and quantifying their current and future carbon (C) stocks is the first step in assessing their potential role in climate change mitigation. Although the FNO forests are unmanaged, the latter task is made more important by growing interest in developing the region’s natural resources, primarily for timber harvesting. In this study, we used a combination of field and remotely sensed observations with a land surface model to estimate forest C stocks in the FNO forests and to project their future dynamics. The specific objective was to simulate historical C stocks for 1901–2014 and future C stocks for 2015–2100 for five shared socioeconomic pathway (SSP) scenarios selected as high priority scenarios for the 6th Assessment Report on Climate Change.

**Results:**

Carbon stocks in live vegetation in the FNO forests remained relatively stable between 1901 and 2014 while soil organic carbon (SOC) stocks steadily declined, losing about 16% of their initial value. At the end of the historical simulation (in 2014), the stocks were estimated at 19.8, 46.4, and 66.2 tCha^−1^ in live vegetation, SOC, and total ecosystem pools, respectively. Projections for 2015–2100 indicated effectively no substantial change in SOC stocks, while live vegetation C stocks increased, accelerating their growth in the second half of the twenty-first century. These results were consistent among all simulated SSP scenarios. Consequently, increase in total forest ecosystem C stocks by 2100 ranged from 16.7 to 20.7% of their value in 2015. Simulations with and without wildfires showed the strong effect of fire on forest C stock dynamics during 2015–2100: inclusion of wildfires reduced the live vegetation increase by half while increasing the SOC pool due to higher turnover of vegetation C to SOC.

**Conclusions:**

Forest ecosystem C stock estimates at the end of historical simulation period were at the lower end but within the range of values reported in the literature for northern boreal forests. These estimates may be treated as conservatively low since the area included in the estimates is poorly studied and some of the forests may be on peat deposits rather than mineral soils. Future C stocks were projected to increase in all simulated SSP scenarios, especially in the second half of the twenty-first century. Thus, during the projected period forest ecosystems of the FNO are likely to act as a C sink. In light of growing interest in developing natural resources in the FNO, collecting more data on the status and dynamics of its forests is needed to verify the above-presented estimates and design management activities that would maintain their projected C sink status.

**Supplementary Information:**

The online version contains supplementary material available at 10.1186/s13021-021-00184-5.

## Background

Forests occupy over 40 million km^2^ globally, accounting for about 30% of total land area, and store about 860 billion tonnes of C in live biomass and dead organic matter pools [51]. The amount of C stored in forests continues to increase, partly offsetting increasing atmospheric CO_2_; forests are the main component of the terrestrial C sink, estimated as of 2018 at 3.5 billion tonnes of C annually or about 30% of total annual emissions [21]. Not surprisingly, forests feature prominently in discussions about climate change and its mitigation [62]. Of particular importance is estimating forest’s future C stocks and whether they will continue to act as a C sink given that climate change is projected to substantially affect forest condition and growth. On the one hand, rising air temperature and atmospheric CO_2_ concentrations can stimulate forest productivity, especially in areas where soil moisture, nitrogen (N), and phosphorous are not a limiting factor [17]. On the other hand, positive effects on productivity may be counteracted by heat-related stress in plants, worsening drought conditions, and changes in disturbance regime [17]. Therefore, quantifying the effects of climate change on forests is needed to develop climate change mitigation strategies.

Canadian forests occupy about 3.56 million km^2^, of which 3.09 million km^2^ are in the boreal zone and the remainder in the temperate zone [53]. Of the total area, 2.3 million km^2^ are covered by managed forests, as defined for the purposes of annual reporting on greenhouse gas (GHG) emissions and removals, while another 1.26 million km^2^ of forested area in northern latitudes are unmanaged [37]. Managed forests are much better studied in terms of their C stocks, due to international reporting obligations as well as historical interest in collecting data and developing tools for timber supply needs, with various components serving as inputs to C balance estimation [35]. The current state of C stocks is much less known for unmanaged forests, and despite recent efforts in data collection (e.g., National Forest Inventory plot grid), major gaps and uncertainties remain unaddressed [35].

**Fig. 1:**
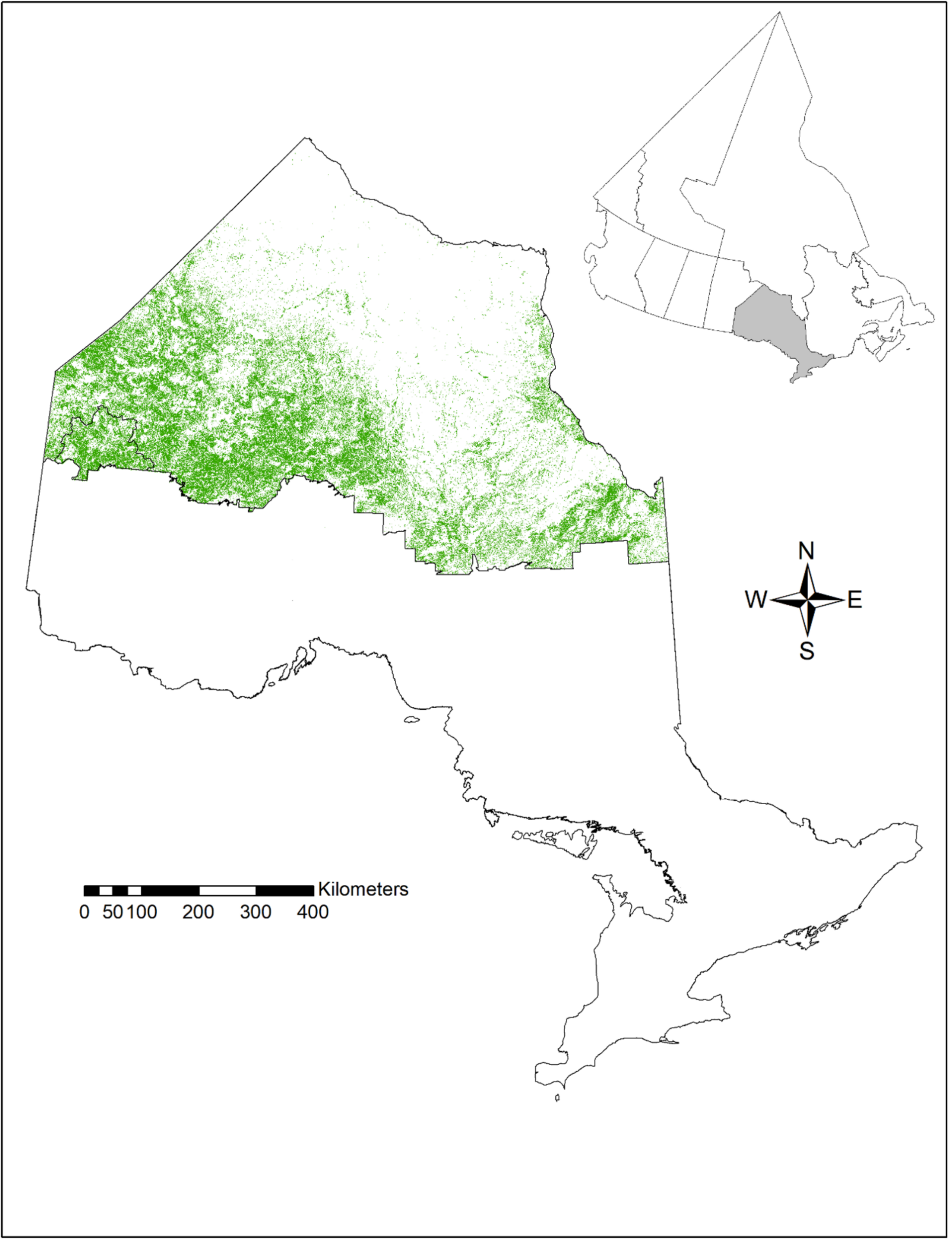
Boundaries of the Far North of Ontario (FNO), Canada, with area in green classified as non-wetland forest. Delineated area in bottom left corner of the FNO is the Whitefeather Forest Management Unit

In Ontario, the unmanaged portion of the forest is in the northern part of the province, usually referred to as the Far North of Ontario (FNO), that lies roughly above the 51st parallel (51°N; see Fig. 1). This area is likely the least studied part of northern forests in North America compared with, for example, Alaska and Quebec, where considerable effort has been focused on the assessment of forest resources and C stocks [41, 45]. Meanwhile, the effects of climate change in the FNO are projected to be more pronounced relative to other parts of Canada; for example, increases in the average winter temperature by the end of twenty-first century are projected to double those in southern Ontario [14].

The first published attempt at estimating current and predicting future C stocks in forests of the FNO was undertaken by [26]. In that study, historical and future C stocks were estimated from 1900 to 2100 for two climate change scenarios (representative concentration pathways RCP4.5 and RCP8.5). The current study builds on [26] by using newly available data sources, correcting winter temperature bias, and expanding future projections to cover the broader spectrum of climate change scenarios. More specifically, the objective was to simulate changes in vegetation and SOC stocks for historical and five shared socioeconomic pathway (SSP) scenarios from the Sixth Assessment Report on Climate Change by the IPCC [42]. The latter projections would provide forest managers and policy makers with benchmark estimates of forest C stocks in the unmanaged forests of Ontario and indicate whether, in the twenty-first century, these forests will act as a C sink or source under possible climate change scenarios.

## Results

**Fig. 2:**
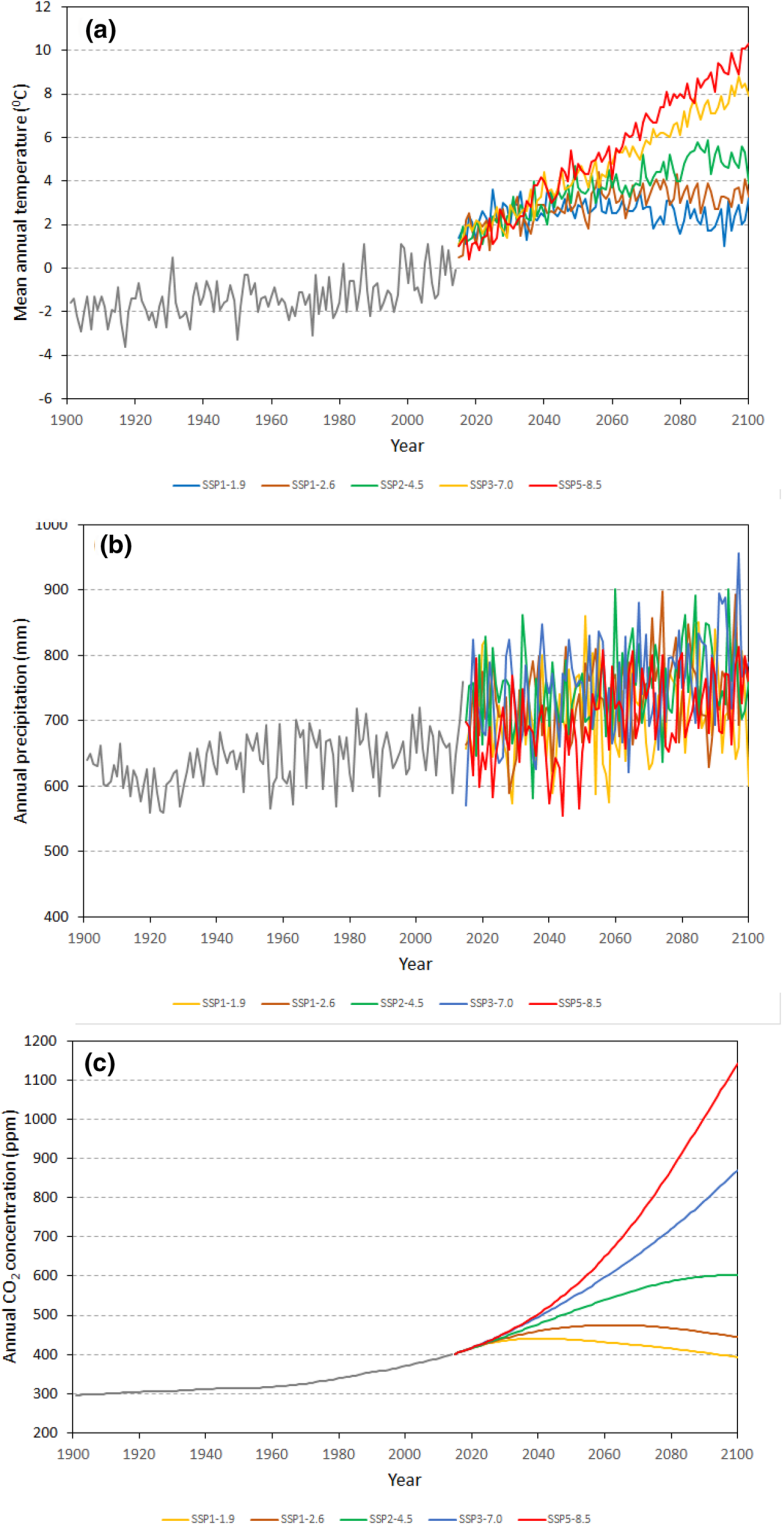
Mean annual temperature (**a**), total annual precipitation (**b**) in the Far North of Ontario, and surface CO_2_ concentration in Northern Hemisphere (**c**) from historical (Climate Research Union observations, [42]) for 1901–2014 and shared socioeconomic pathway scenarios for 2015–2100 (CanESM5 projections, [42])

Carbon stocks were simulated for 120,634 km^2^ of area classified in the land cover map of the FNO as non-wetland coniferous, deciduous, or mixedwood forest (Fig. 1). Hereafter this area is referred to as the FNO forested area or the FNO forests; see "Methods" for study area description. Simulations were performed for historical data for 1901–2014 and five SSP scenarios for 2015–2100 (SSP1-1.9, SSP1-2.6, SSP2-4.5, SSP3-7.0, and SSP5-8.5) Mean annual temperature, total annual precipitation, and atmospheric CO_2_ concentration for the simulation periods are shown in Fig. 2, while summary statistics are presented in Table 1.

**Table 1:** Summary statistics on annual air temperature and annual total precipitation during 2015–2100 for the Far North of Ontario for five shared socioeconomic pathway (SSP) scenarios

Scenario	SSP1-1.9	SSP1-2.6	SSP2-4.5	SSP3-7.0	SSP5-8.5
Annual air temperature (°C)					
Mean (°C)	2.50	2.89	3.62	4.77	5.32
Min (°C)	1.91	1.64	1.54	1.78	1.13
Max (°C)	2.90	3.68	5.37	8.07	9.54
Annual total precipitation					
Mean (mm)	707.8	735.8	752.4	754.0	702.4
Min (mm)	663.6	680.0	715.5	697.4	634.5
Max (mm)	764.8	793.9	811.1	836.5	752.5

Minima and maxima for both temperature and precipitation are estimated for 10-year moving averages

**Fig. 3:**
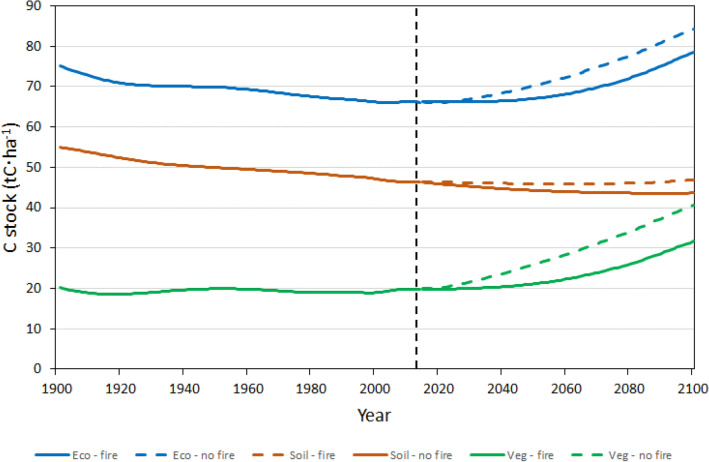
Historical (1901–2014) and projected (2015–2100) for SSP2-4.5 scenario carbon density in live vegetation (green lines), soil organic carbon (SOC; brown lines), and total ecosystem (blue lines) in the forests of the Far North of Ontario (FNO). Solid and dashed lines correspond to projections with and without fire, respectively; vertical dashed line indicates the end of historical simulation

**Fig. 4:**
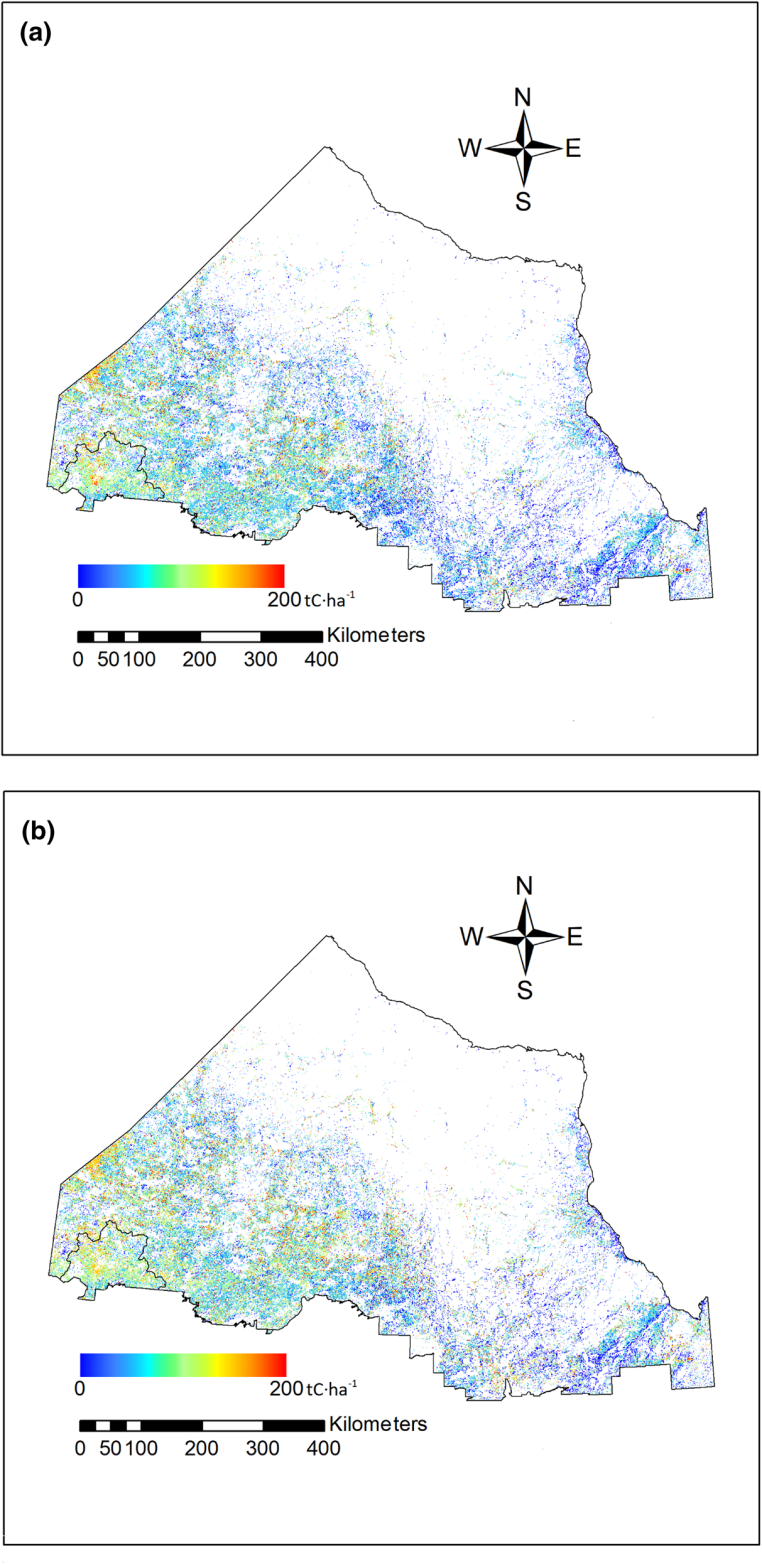
Map of total ecosystem carbon density for the forested area of the Far North of Ontario (FNO) for **a** 2015 and **b** 2100 for SSP2-4.5 scenario with fires

Simulated historical (1901–2014) C stocks in live vegetation, soil, and total ecosystem are presented in Fig. 3, along with projected (2015–2100) C stocks for SSP2-4.5 scenarios with and without wildfires; maps of spatial distribution of C stocks density across forested area of the FNO for 2015 and 2100 simulated for SSP2-4.5 scenario wildfires are shown in Fig. 4. The latter scenario was selected for presentation of the results because it is a “middle-of-the-road” one among the five scenarios used in this study, with the mean 2015–2100 annual air temperature and atmospheric CO_2_ concentration of 3.62 °C and 522 ppm, respectively (see Table 1 and Fig. 2). As seen in Fig. 3, between 1901 and 2014, C stocks in live vegetation in the FNO forests remained relatively stable, ranging from a minimum of 18.3 tCha^−1^ in 1918 to a maximum of 20.2 tCha^−1^ in 1901. Soil organic carbon stocks, on the other hand, steadily declined from 54.9 tCha^−1^ in 1901 to 46.3 tCha^−1^ in 2014; here and throughout the text SOC includes organic C in the upper 1 m of soil and dead organic matter (DOM) pools such as litter, downed dead wood, and standing dead trees. Consequently, total forest ecosystem C stocks also declined from 75.2 tCha^−1^ in 1901 to the minimum of 65.9 tCha^−1^ in 2002 and then rebounded slightly to 66.1 tCha^−1^ in 2014 (Fig. 3).

As expected, model runs showed strong effects of wildfires on the projected C stocks. In the absence of wildfires, live vegetation C stocks doubled from 19.9 tCha^−1^ in 2014 to 40.7 tCha^−1^ in 2100; simulation of wildfire activity reduced this increase almost by half, to 31.7 tCha^−1^ in 2100 (Fig. 3). Simulating wildfires had the opposite effect on SOC, which effectively showed no change from 46.3 tCha^−1^ in 2014 to 46.9 tCha^−1^ in 2100 with wildfires, and decreasing to 43.7 tCha^−1^ in 2100 in the absence of wildfires. Summation of live vegetation C and SOC resulted in forest ecosystem stocks increasing from 66.2 tCha^−1^ in 2014 to 84.3 tCha^−1^ and 78.6 tCha^−1^ in 2100 without and with wildfires, respectively (Fig. 3).

**Fig. 5:**
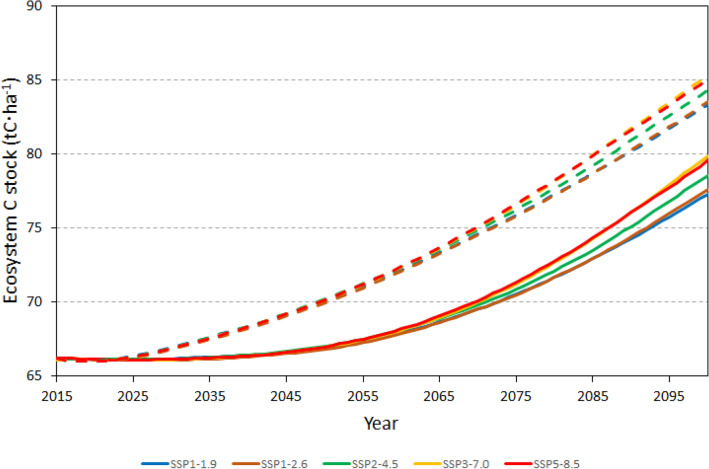
Projected (2015–2100) total forest ecosystem carbon stock density in forests of the Far North of Ontario (FNO) for five climate change scenarios: SSP1-1.9 (blue); SSP1-2.6 (brown); SSP2-4.5 (green); SSP3-7.0 (yellow); SSP5-8.5 (red). Solid and dashed lines correspond to projections with and without fire, respectively

The patterns presented in Fig. 3 were consistent among all five climate change scenarios; changes in total forest ecosystem C stocks for the five scenarios, with and without simulation of wildfires, are presented in Fig. 5. In all scenarios, total forest ecosystem C stocks dropped slightly in 2020–2025 losing the maximum of 0.14 in SSP1-2.6 scenario. Subsequently, the stocks steadily increased until 2100, reaching values between 77.3 tCha^−1^ and 79.9 tCha^−1^ (Fig. 5). Thus, by the end of the century, total forest ecosystem C stocks in the FNO are projected to increase by 16.7–20.7% relative to 2015. The results of simulation with and without wildfires for all climate change scenarios are shown in the Additional file 1.

**Fig. 6:**
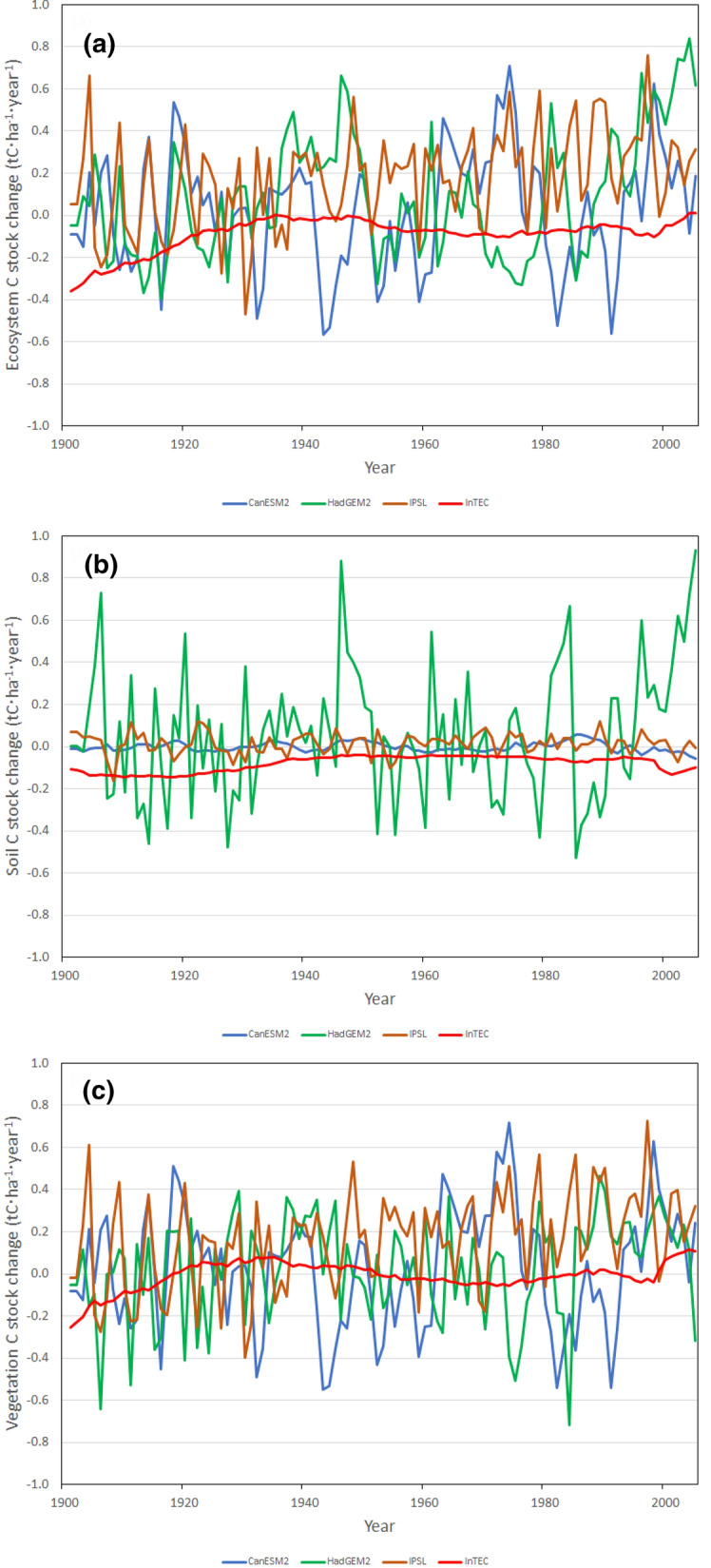
Historical (1901–2005) carbon stock changes in **a** total ecosystem, **b** soil and **c** live vegetation in forests of the Far North of Ontario (FNO) simulated by InTEC (this study) and three Earth System Models (CanESM2, HadGEM2, and IPSL)

**Fig. 7:**
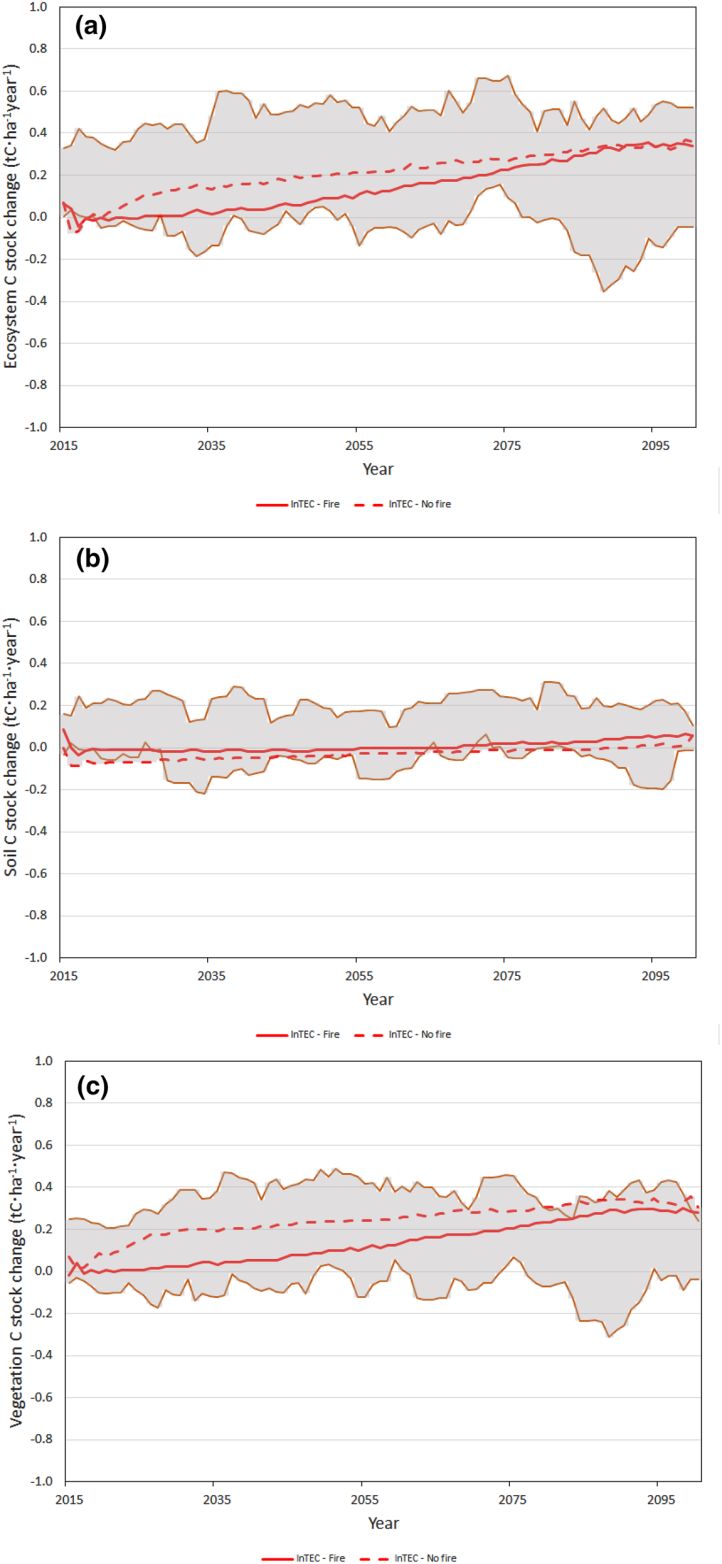
Projected (2015–2100) carbon stock changes in **a** total ecosystem, **b** soil and **c** live vegetation in forests of the Far North of Ontario (FNO) simulated by InTEC (this study). Highlighted in grey is the area between five-year moving average of maxima and minima from six scenarios simulated by three Earth System Models (CanESM2, HadGEM2, and IPSL); see text for more detail

Figure 6 shows forest ecosystem, vegetation, and soil C fluxes (i.e., annual changes in C stocks) simulated for the historical period 1991–2005 in this study and by three Earth System Models (ESMs): CanESM2, HadGEM2, and IPSL. Results are shown for 1991–2005 because it is the overlapping period between InTEC and the three ESM simulations forced by observed changes in atmospheric composition and land cover. Figure 7 shows C fluxes for 2015–2100 projected in this study for SSP2-4.5 scenario and the averages of five-year moving minima and maxima over six scenarios projected by the three ESMs and calculated as follows. For a given year and for each model-scenario combination, a minimum and maximum are estimated over a five-year period centering on the given year, and the average minimum and maximum are then calculated for the six scenarios. The results for scenario SSP2-4.5 from this study are shown in Fig. 7 since this scenario is most similar to RCP4.5 scenarios used in the ESMs. The fluxes projected by the ESMs for all six scenarios are shown in Fig. S1.

## Discussion

Live vegetation C stocks estimated for 2014 were within the range of values reported for northern boreal forests, albeit at the lower end. Globally, live vegetation C in the boreal forest biome is estimated at 46.7 tCha^−1^ as of 2007 [51]. However, it is not uncommon to see much lower estimates, especially for the northern boreal forest. For example, Gower et al. (2001) reported values of 12.8, 16.1, 99.9, and 15.3 tCha^−1^ in black spruce (*Picea mariana* (Mill.) BSP)-dominated stands in Alaska, USA, aged 51, 55, 130, and 200 years old, respectively. Other estimates of mean live tree biomass for the same region include 26.0 tCha^−1^ for three black spruce stands ranging in age from 160 to 200 years [56] and 25.5 tCha^−1^ for four black spruce-dominated stands with an average age of 95 years [13]. Schvidenko and Nilsson [60] estimated live vegetation C stocks at 23.9 tCha^−1^ for forest tundra in the northern and sparse taiga bio-climatic zone in Russia while [45] used two different methods to arrive at estimates of 18.8 and 25.4 tCha^−1^ for total aboveground vegetation in the northern and southern boreal zones of Quebec, respectively.

Similarly, our estimate of SOC is at the low end of values reported in the literature. For example, Schulze et al. [58] estimated the average SOC content of a chronosequence (28–383 years) of six Scots pine (*Pinus sylvestris* L.) stands in Central Siberia, Russia, at 37.8 tCha^−1^, of which 18.2 tCha^−1^ were in mineral soil and 19.6 tCha^−1^ in DOM pools (forest floor, downed dead wood, and standing dead trees). In a similar study in Central Siberia, the average mineral soil and DOM C stocks in four chronosequences of Scots pine stands (total of 22 stands) was estimated at 15.6 tCha^−1^ and 31.8 tCha^−1^, respectively [73]. In a 68-year-old Norway spruce (*Picea abies* L.H.Karst) stand growing in the northern boreal zone in the European part of Russia, C stocks in mineral soil and dead organic surface layers were estimated at 43.5 tCha^−1^ and 7.8 tCha^−1^, respectively [32]. In the latter three studies [32, 58, 73], C in mineral soil was measured to 0.5 m depth. However, based on the analysis of nearly 17,000 forest soil measurements, De Vos et al. [16] concluded that the top 0.5 m accounts for 77% of the total C content in 1 m of mineral soil. Thus, if we expand the estimates from [32, 58, 73] by applying a correction factor of 1.3 estimated in [16], the resulting C content in the DOM pool is about the same as that estimated in this study.

It is worth noting that, in 2014, C stocks in live vegetation in the FNO forest accounted for 34.4% of total forest ecosystem C stocks. This number is similar to the estimated fraction of live vegetation C stocks in several other studies, e.g., 30.1% in [34] or 33.7% in [9]. In other words, our simulated estimate of the ratio of live vegetation C to SOC is within the range of values reported in the literature.

Live vegetation C stocks simulated in this study remained relatively stable during 1900–2015. These results are consistent with the conclusions reached by [8]; the authors of the latter study analysed more than 20,000 sample plots in five states across the United States and did not find substantial increases in forest growth rates during the twentieth century. In a large-scale study that included permanent sample plots in five provinces, no significant trends were observed in the rate of biomass change in eastern Canada (Ontario and Quebec) from mid-seventies of the twentieth century to the early years of this century [39]. In an unrelated large-scale study based on the analysis of tree-ring width data covering about 60 years starting in 1950, significant changes were not found in productivity of black spruce and jack pine (*Pinus banksiana* Lamb.) forests growing in the Boreal Shield West region of Canada, which includes the FNO [25].

During the same historical period, SOC stocks steadily declined, diminishing nearly 16% by 2014 relative to 2001. This decline is likely caused by two factors. The first one is an increase in soil respiration and rate of organic matter decomposition caused by rising mean air temperature that, during 1901–2014, on average increased by 1.5 °C in the FNO [26]. For example, a heating experiment in a red spruce (*Picea rubens* Sarg.)-dominated forest in Maine, USA, showed an increase in litter decay as a result of warming, and consequently a significant loss of litter mass and C content [57]. In another study in mixed hardwood forest in Massachusetts, USA, soil C losses were recorded as a result of increased temperature, primarily due to microbial respiration associated with soil organic matter decay that was responsible for over three quarters of total annual CO_2_ efflux from soil [43]. The second factor is the above-discussed stability of live vegetation stocks that is a source of C transfers to SOC pool. The combination of increasing efflux and constant influx resulted in the steady loss of SOC simulated in our study.

Trends in historical C stocks and their fluxes simulated in this study are similar to those simulated for the FNO using three ESMs (Fig. 6 and Additional file 1). Estimated as a slope of a simple linear regression, the trend in historical vegetation C stocks (0.0025) and C fluxes (0.0009) are within the ranges for trends in stocks (0.0011–0.0159) and fluxes (0.0009–0.0029) of those simulated using the ESMs. The same is true for the trends in soil C fluxes (0.0007 in this study vs. 0.0–0.0019 for ESMs) and in ecosystem C fluxes (0.0015 in this study vs. 0.0010–0.0035 for ESMs). The only historical trends projected in this study that fall out of those simulated by the ESMs are those for soil C stocks (− 0.0685 in this study vs. 0.0–0.0014 for ESMs) that in turn dictate the negative trend for ecosystem C stocks (-0.0662 vs. 0.0012–0.0173 for ESMs). However, vegetation C stocks are better studied and quantified than those for soil, and vegetation C stocks and fluxes simulated in this study closely agree with both the empirical observations [25, 39] and simulations using the ESMs. This finding, combined with the above-discussed interplay between vegetation and soil C stocks serves as a verification of historical C stocks and fluxes simulated in this study for the FNO.

Simulations of C stocks during 2015–2100 were performed for five climate change scenarios based on the so-called shared socioeconomic pathways (SSPs) combined with various mitigation efforts [24]. The SSPs describe how the world might evolve based on socioeconomic factors such as population, economic growth, education, urbanisation, and the rate of technological development [55]. The five scenarios used in this study were selected as “high-priority scenarios” for the Sixth Assessment Report on Climate Change by the IPCC and included: three scenarios—SSP1-2.6, SSP2-4.5, and SSP5-8.5—approximately corresponding to the previous generation scenarios RCP 2.6, RCP 4.5, and RCP 8.5, respectively; “medium–high” scenario SSP3-7.0; and SSP1-1.9, which most closely reflects a 1.5 °C target under the Paris Agreement [42]. For more detail on the development of SSPs and for the so-called narratives (i.e., textual description of how the future might unfold in terms of broad societal trends) of SSPs selected for this study the reader is referred to [55].

Vegetation C stocks in the twenty-first century increased in all climate change scenarios. Similar trends have been simulated in other studies. For example, simulations of total land C (vegetation plus SOC) in British Columbia, Canada, projected an increase in C stocks from 2005 to 2050 by 12% and 16% (compared to 2005) for the lowest (RCP2.6) and highest (RCP8.5) scenarios, respectively [3]. Changes modelled in our study for the same period are lower, likely because SOC stocks are projected to remain relatively stable throughout the twenty-first century. In another North American study, changes in terrestrial C stocks in Alaska, USA, were simulated by two general circulation models for three SRES scenarios (A1B, A2, and B1) [23]. The authors of the latter study projected an increase in upland vegetation C stocks in all six scenarios ranging from 12 to 24%. Modelling studies for boreal forests in Europe also indicated positive trends in C stocks and productivity. Increases in net primary productivity of the European boreal zone are projected by the end of the twenty-first century for A1B and B1 scenarios generated by three general circulation models [54]. Similar results were produced by [33] who used a variety of climate change scenarios to evaluate sources of uncertainty in projections of the primary productivity in boreal forests of Finland and concluded that productivity was likely to increase by the end of the century.

The changes in C stocks projected in this study are consistent with those simulated using the three ESMs. For example, forest ecosystem C stocks are projected to increase by 18.7% during 2015–2100 in the SSP2-4.5 scenario; respective changes simulated by the ESMs range from − 7.3% (for the CanESM2-Climate scenario) to 28.8% (for the HadGEM2-CO_2_ scenario). A comparison of future linear trends in C stocks (as was done above for the historical period) would not be informative due to the non-linear nature of C stock dynamics (Fig. 5). The C stock fluxes, however, also indicate good agreement between the results of this study and the CMIP5 project: as seen from Fig. 7, for both SSP2-4.5 scenarios (with and without wildfires) the C stock fluxes for all three pools (ecosystem, soil, and vegetation) are within the range of minima and maxima simulated using the three ESMs.

Results from several ESMs indicate that rising atmospheric CO_2_ will promote C uptake by both plant biomass and soil organic matter [65, 67]. These results seem to be based on the concept of increased vegetation growth resulting in higher input to and therefore an increase in soil C. However, the meta-analysis of 53 experiments undertaken by [67] indicated that atmospheric CO_2_ enrichment stimulates both the input and the turnover of C in soil, and therefore does not significantly affect soil C content. In other words, increased CO_2_ stimulates plant growth and C input to soil; however, the response is counteracted by the microbial response, reducing the net effect on soil C stocks [67]. This finding is echoed by [65] who state “there is both empirical and theoretical evidence that increases in soil inputs, especially under elevated CO_2_, may have little effect on soil organic carbon stocks.” Results of another meta-analysis of 49 field experiments, located across North America, Europe, and Asia, suggest that the effect of warming depends on the amount of initial soil C stock: effects are negligible for sites with small initial C stock but occur beyond the stock values of 200–400 tCha^−1^ and are considerable in soil with stocks higher than 600 tCha^−1^ [15]. The area simulated in our study belongs to the former category of low initial SOC stocks; hence, stocks are projected to remain relatively stable throughout the simulation period. As stated by [15], in ecosystems with low initial SOC losses resulting from increased decomposition rate due to warming, changes are relatively minor and can be offset by concurrent increases in vegetation growth and soil C stabilization; however, in areas with large initial SOC, increased decomposition occurs faster than C accumulation from enhanced vegetation growth and therefore leads to substantial C losses to the atmosphere [15].

One of the predicted outcomes of the future C stock dynamics is the change in allocation of C between live vegetation and SOC pools (Fig. 3). By the end of the historical simulation (i.e., in 2014), live vegetation accounted for 30% of total ecosystem C. That fraction increased by 2100 to about 40%, ranging from 39.3% to 41.8% among scenarios. This shift in C allocation with the increasing temperature and atmospheric concentration of CO_2_ has been observed in empirical studies. For example, in a CO_2_-enrichment experiment in Massachusetts, USA, a deciduous forest stand increased the share of C stored in live vegetation while losing some of the SOC stock [43]. The shift in relative distribution of C between forest floor and aboveground vegetation with increasing soil temperature has been reported in a comparative study of C allocation in nine black spruce stands in boreal forests of Canada and the United States [69]; similar change in relative distribution of C with increasing air temperature was observed in Scots pine forests in Europe [69].

Previous simulations demonstrated the effects of strong winter warmth biases inherent in ESMs [26]. The latter biases may lengthen the growing season, which in turn would result in an increase in vegetation C stocks and consequently SOC. For the current simulations, the winter biases were corrected for all climate variables at a monthly time scale with the linear scaling bias correction method using observed and simulated data from overlapping years (2015–2018). Comparison between the closest scenarios in this study and [26] (SSP5-8.5 and RCP8.5 + historical fire scenarios, respectively) showed that without winter bias correction, by the end of the twenty-first century, live vegetation, SOC stocks, and total ecosystem C stocks were overestimated by 15.5%, 3.0%, and 7.9%, respectively. This finding is consistent with the effect of bias correction on changes in C pools reported by [2] and emphasizes the importance of bias correction for more realistic prediction of C stocks.

Total C stocks in forests of the FNO in 2014 were estimated at 798.6 million tonnes, of which live vegetation and DOM contained 239.2 and 559.4 million tonnes, respectively. For comparison, the total amount of C in 29.4 million hectares of Ontario’s forests managed for timber production has been estimated at 4,719.2 million tonnes, for the average density of 160.5 tCha^−1^ [11]. The lower C density in FNO forests may be caused by differences in species composition, climatic conditions, and the lack of active wildfire suppression measures applied in the managed forest. Also, since we excluded areas classified as treed wetland, our results may underestimate C storage in FNO forests. It is also possible that some of the forests included in our study are on peat deposits rather than mineral soils [26]. Overall, this uncertainty underscores the need for collecting data on the condition and dynamics of the FNO forest; primarily forest inventory information, which is lacking for almost the entire FNO, but also sample plots that would allow assessment of changes in forest composition and characteristics over time.

Simulations with and without wildfires illustrated the effects of wildfires on C stocks in the FNO forests (Fig. 3). In the absence of wildfires, live vegetation C stocks in SSP2-4.5 scenario increased twofold by the end of twenty-first century; the increase was much smaller when wildfires were simulated, reflecting losses of C to combustion emissions and transfers to SOC pool [35]. The latter transfers result in wildfires having the opposite effect on SOC stocks that are higher in the scenario with wildfires (Fig. 3). Simulations without wildfires also corroborated the previously discussed effect of increasing air temperature on soil respiration. In the absence of wildfires and other disturbances, soil C stocks are primarily controlled by C inputs from the vegetation pool and C losses to the atmosphere due to soil respiration. As vegetation C stocks increase, C inputs into the soil pool increase [12] so to maintain stable soil C stocks, these inputs must be offset by increases in soil respiration (Fig. 3). Thus, simulations without fire indicate that soil respiration increases with air temperature which is consistent with empirical observations [43, 57].

Simulation of wildfires also sheds light on their relative contribution versus that of climate change to changes in C stocks of the FNO forests (Figs. 3 and 5). The difference in projected live vegetation C stocks between scenarios with and without wildfires may be attributable to changes in climatic variables and CO_2_ fertilisation. This attribution is supported by the pattern of C stock dynamics during the first half of the twenty-first century. Without wildfires, live vegetation (Fig. 3) and total ecosystem (Fig. 5) C stocks start increasing immediately after 2015 because wildfire simulation stops at the end of historical period, leading to increases in forest age. However, in the scenario with wildfires forest age is unlikely to increase, and changes in C stocks become noticeable much later since it takes longer time for the effects of gradual changes in climate and atmospheric CO_2_ concentration to become pronounced. It is worth noting that, by the end of simulation period, the rate of increase in ecosystem C stocks in simulations with wildfires fire matches or exceeds that in scenarios without wildfire (Fig. 5). This effect is similar to previously reported simulations in which increases in the average annual fire-burned area do not reduce forest ecosystem C stocks when the effect of CO_2_ fertilization is accounted for [4, 26]. While the latter result may seem counterintuitive, the proposed explanation lies in wildfire-caused changes to forest age structure: fires replace older slow growing forest stands with young ones that can take advantage of the increasing atmospheric CO_2_ concentration. The resulting accelerated biomass growth combined with slow decomposition of fire-transferred matter to SOC pools is sufficient to compensate for C losses due to direct combustion emissions.

One of the limitations of this study is the assumption of equal probability of burning for all pixels classified as forest (with the exception of forests younger than 11 years). Meanwhile, indications are that individual wildfire events may exhibit selectivity for species composition and forest age. For example, Bernier et al. [6] in a Canada-wide study of wildfires during 2002–2011 concluded that probability of burning was significantly higher in forests older than 30 years as well as in forests dominated by conifers (vs. broadleaved stands). Another limitation is the lack of dynamic representation of species composition; albeit not unique in the modelling literature (e.g., see [3]), the assumption of fixed spatial distribution of forest types does not reflect possible effects of climate change on this distribution. For example, studies in Alaska (another large area of unmanaged northern boreal forests in North America) suggested that changes in the wildlife frequency and intensity have the potential to increase the extent of deciduous forest cover at the expense of the conifers [23, 30]. More research is needed to assess how total forest C stocks would be affected by these two potentially counterbalancing phenomena, i.e., higher probability of wildfires targeting older conifer stands that contain higher C stocks vs. reduced probability of wildfires resulting from the increasing fraction of deciduous forests.

Climate change is projected to increase the burned area in the FNO. For example, [20] estimated the annual burned area for a scenario corresponding to a threefold increase in the atmospheric concentration of CO_2_, with 1.5–2.0 increases projected in the western part of the FNO (containing most of the FNO forests) and 2.1 increase in the eastern part relative to levels registered during 1959–1997. Boulanger et al. [7] projected the annual burned area to increase less than twofold and fourfold in the western and eastern parts of the FNO, respectively; the latter increases were projected to occur during 2071–2100 in the A2 scenario [7]. These predictions are likely to be at the upper limit of possible changes in the fire-burned area since triple-CO_2_ and A2 scenarios are close to the most aggressive RCP8.5 and SSP3-7.0-SSP5-8.5 scenarios [42, 68]. Unfortunately, neither paper [7, 20] contained the models used in their predictions. In the precursor to this study [26], future fires were simulated using equations from [5]. However, this meant using equations with relatively low explanatory power (R^2^ < 0.5) and extrapolating them to the parts of the FNO for which no equations have been estimated. As noted in [5], this is one of the areas in eastern Canada where developing fire models driven by fuel moisture and temperature is more challenging due to the influence of large water bodies (Hudson and James bays). In the absence of reliable models predicting future fire regime in the FNO for various climate change scenarios, we decided on the conservative approach of simulating the historical rate of annual burned area.

Finally, future emissions from wildfires may also be affected by increases in proportion of combusted soil material. For example, a study in black spruce-dominated stands in Alaska indicated that the depth of burning in the ground layer of biomass (including moss, litter, organic soils, and peat) increased with the length of the wildfire season [66]. Similarly, studies conducted in boreal forests of the Northwest Territories (Canada) showed that C soil combustion depended on the forest stand’s landscape position and age [70, 71]. As with the above-discussed ambiguity of wildfires’ forest type selectivity and possible shifts to more deciduous-dominated forests, more studies are needed to assess whether higher growth rate in young stands due to CO_2_ fertilization can overcome C losses from the increased spatial extent and depth of burning of future wildfires. The latter is important as the province of Ontario, among many other jurisdictions, attempts to reduce its GHG emissions [44]. Currently, the FNO forests fall in the category of unmanaged and therefore, according to international rules, their C balance would not count towards GHG emission reduction targets. However, despite the current surplus in harvestable timber in the managed forests of Ontario [50], the interest in developing natural resources of the FNO, primarily timber harvesting, is growing [48]. Harvesting is the main forest management activity affecting forest C dynamics in North American forests by reducing their C stocks and emitting C to the atmosphere [17]. Although various management activities could increase forest uptake of atmospheric C and decrease emissions in the forest sector [37], it would be challenging to develop resource management in the FNO while maintaining the projected C sink status of its forests. However, regardless of possible management activities, the principal need is the above-mentioned data collection in the FNO forests that would allow verification of their current state and reduce uncertainty about their future dynamics.

## Conclusions

We simulated historical (1901–2014) and future (2015–2100) C stocks in forests of the FNO. These simulations improved on previous results by using a better-defined land base and a larger number of more up-to-date climate change scenarios. Simulated live vegetation C stocks were relatively stable during the historical period, while SOC stocks steadily declined, losing about 16% of their initial amount by the end of the historical period. Consequently, total forest ecosystems C stocks were also reduced by about 12%. Carbon stock estimates at the end of historical simulation period were at the lower end, but within the range, of values reported in the literature for northern boreal forests. These estimates may be treated as conservatively low; the area included in the estimates is poorly studied and it is possible that some of the forests are on peat deposits rather than mineral soils. Future C stocks were projected for five SSP scenarios selected as “high-priority scenarios” for the Sixth Assessment Report on Climate Change by the IPCC. Soil organic carbon stocks were projected to remain relatively constant from 2015 to 2100; live vegetation C stocks, however, were projected to increase, especially in the second half of the twenty-first century. These results were consistent among all five simulated climate change scenarios. The results were improved by applying a correction to winter warmth bias; in the absence of this correction, vegetation, SOC, and total ecosystem C stocks would be overestimated by up to 15.5%, 3.0%, and 7.9%, respectively. By and large, during the projected period forest ecosystems of the FNO are likely to act as a C sink. In light of growing interest in developing natural resources in the FNO, collecting more data on the status and dynamics of its forests is needed to be able to verify the above-presented estimates and design management activities that would maintain their projected C sink status.

## Methods

Methods and several input data sets repeat those used in the precursor to this study [26]. Therefore, here we briefly describe models and input data used in the study, with more attention to the novel elements. The latter include new landcover data, updated version of historical climate data, and new projections of future climate data corresponding to five SSPs (as opposed to only two scenarios of an older vintage used in [27]). Future climate data were corrected for winter biases the effects of which were evident in the previous simulations [27].

The FNO is an area of 439,756 km^2^ located approximately to the north of 51 °N and bound from the west and east by Manitoba and Hudson and James bays, respectively [47] (Fig. 1). The FNO overlaps two ecozones: Hudson Plains and Boreal Shield. Forested areas are located mostly in the Boreal Shield since flat terrain and poor drainage of the Hudson Plains has resulted in the largest contiguous wetlands in the world. Tree species composition in the FNO is typical of northern boreal forests of Canada, with black spruce being the dominant species, particularly on lowland sites, along with white spruce (*Picea glauca* (Moench) Voss), jack pine (*Pinus banksiana* Lamb.), trembling aspen (*Populus tremuloides* Michx.), tamarack (*Larix laricina* (Du Roi) K. Koch), and white birch (*Betula papyrifera* Marsh.) [26]. This study includes land cover cells classified as non-wetland coniferous (104,426 km^2^), deciduous (5,770 km^2^), and mixedwood (10,438 km^2^) forests totalling 120,634 km^2^ [27]; areas classified as treed wetland were not included. The long-term (1901–2015) average mean annual air temperature and total annual precipitation are − 1.3 °C and 640 mmyear^−1^, respectively. Forests in the study area are classified as unmanaged. In 2009, a fraction of the study area along the southwest boundary of the FNO was designated an addition to the forests managed for timber production (Fig. 1). A forest management plan for 6,270 km^2^ of forested area in the newly formed Whitefeather Forest Management Unit (FMU) was developed in 2012. However, to date no harvesting or other management activity has occurred in the Whitefeather FMU [49]; therefore, the entire forested area included in this study is considered unmanaged.

The forest C cycle was modelled using the Integrated Terrestrial Ecosystem Carbon Cycle (InTEC) model developed to simulate C balance in Canada’s forests by integrating effects of disturbance and non-disturbance factors such as climate, CO_2_ concentration, and N deposition [12]. Complete theories and formulations of the InTEC model are documented by [10, 12, 31]. In the model, carbon balance is simulated using 13 C pools, four of them describing vegetation (foliage, stem, fine root, and coarse root), and nine for SOC (surface structural litter, soil structural litter, woody litter, surface metabolic litter, soil metabolic litter, surface microbial, soil microbial, slow soil organic matter C pool, and passive soil organic matter C pool); simulated SOC pools account for C content to one m depth. The InTEC model consists of three components: a canopy-level photosynthesis module for simulating net primary productivity (NPP), a module for simulating soil C and N dynamics, and a hydrological module for simulating soil moisture and temperature.

The soil C and N dynamics module is based on the CENTURY model [52] modified to account for multiple soil C pools; temporally and spatially varying N depositions; the effects of drainage, soil temperature, and moisture on decomposition rate; and climatic and C pool size effects on N fixation. The hydrological module, parameterized based on fractions of sand, clay, silt, and organic matter and vegetation properties, simulates soil water content and temperatures of three soil layers at monthly time steps needed to quantify decomposition rates of soil C pools and soil water stress effects on photosynthesis. The photosynthesis module was developed from a canopy-level Farquhar’s leaf biochemical model using a temporal and spatial scaling scheme [12, 19]. The module quantifies the integrated effects of changes in stand age, climate, and CO_2_ and N deposition since the preindustrial period on the interannual variability of NPP to progressively calculate annual NPP from an initial NPP value. The NPP value in a reference year (we used 2004 because that was the year all spatial data sets were available), simulated at daily time steps using the Boreal Ecosystem Productivity Simulator (BEPS) [38], was the benchmark used to tune the initial NPP value. For each pixel, the initial NPP value was repeatedly adjusted until the difference between NPP simulated by InTEC and the benchmark output from BEPS in the reference year (i.e., 2004) was less than 1%. Collection of the field data used to validate the BEPS reference NPP validations and development of NPP-age curves are described in detail in [27].

To initialize various C pools, InTEC assumes them to be in a steady state before 1901 for stands disturbed after 1901 or in the year before the most recent disturbance for stands undisturbed after 1901. The initialization was run until the C pools reached a steady state in which the absolute value of the ecosystem net C balance became smaller than 2% of the initial NPP, using as the latter the NPP and stand age in 1901 along with mean climatic conditions in 1901–1910 to initialize C pools in vegetation and SOC. The sizes of the various C pools were estimated by solving a set of differential equations that consider the interaction among pools under steady state C dynamics [10].

All wildfires simulated by InTEC are assumed to be stand-replacing, i.e., causing complete stand mortality. For both historical and projected simulations, the constant number of burned cells is randomly allocated to all forest stands older than 11 years; the probably of getting burned by a wildfire does not depend on forest type and age. Once burned, a fraction of biomass and soil C is emitted to the atmosphere while the rest of biomass C is transferred to the soil pools. The amount of directly emitted C includes 100% of foliage, 100% of surface structural and surface metabolic litter, and 25% of stem wood; the latter fraction accounts for the losses of primary and secondary branches and part of the boles. Dead biomass C remaining after fire is transferred to woody litter and surface metabolic and structural litter pools, and its decomposition is assumed to start the year after fire disturbance. The model does not consider fire intensity and hence does not account for the effect of the latter on C losses. The forest disturbed by wildfires regenerates without a forest cover type change in the second year after a disturbance.

Inputs to the InTEC model included spatial data sets of climatic variables, N deposition, soil texture, drainage, digital elevation model, land cover, leaf area index (LAI), forest stand age, reference NPP, and fire disturbance; the spatial data sets covered the entire FNO forest area. Prior to model runs, all spatial data sets were interpolated to 500 m resolution, the highest spatial resolution of the remote sensing input data sets. Four new data sets were compiled for the current study (compared to [26]), namely: new land cover data, new version of historic climate data (1901–2014), future climate data (2015–2100), and future fire data (2015–2100). A brief description of input data sets is given below.

Land cover data were obtained from the Far North Land Cover, version 1.4 [46]. The initial 24 classes included in the data set were aggregated into six classes (i.e., water, wetland, treed wetland, tundra heath, coniferous forest, deciduous forest, and mixedwood forest). Only non-wetland coniferous, deciduous, and mixedwood forests were considered. The area classified as wetland and treed wetland was excluded because processes controlling C stock dynamics in these ecosystems differ from those in forests [22] and are not simulated by the InTEC model. Gridded monthly climate data, including mean air temperature, water vapour pressure, and precipitation at 500 m resolution for the period 1901–2014 were interpolated bilinearly from the 0.5° global data set produced by the Climate Research Unit, version CRU TS v. 4.03 [29]. The monthly incoming shortwave radiation data for 1901–2014 were obtained from 20th Century Reanalysis V3 data set provided by the NOAA/OAR/ESRL PSL, Boulder, Colorado, USA [61].

The future climate data were from the Canadian Earth System Model (CanESM5) prepared for the sixth phase of the Coupled Model Intercomparison Project (CMIP6) that is under the auspices of the World Climate Research Programme. Future climate data for five scenarios (SSP1-1.9, SSP1-2.6, SSP2-4.5, SSP3-7.0 and SSP5-8.5) were downloaded from [72]. The CanESM5 data were interpolated bilinearly from the 2.8^o^ × 2.8° original spatial resolution to 500 m. Previous simulations demonstrated the effects of strong winter warmth biases inherent in all Earth System Models [26]. For the current simulations, the winter biases are corrected using the so-called delta change and linear scaling bias correction methods [40]; albeit relatively simple, they have been shown to perform well at monthly scale [59]. In our study, the delta change and linear scaling bias correction methods were applied using the observed and simulated data for 2015–2018. For each variable, the overlapping period (2015–2018) provided four years or 48 monthly data points that were used to derive a correction factor. The corrections, based on the difference in the mean monthly value of observed and simulated temperature and on the ratio of the mean monthly value of observed divided by simulated climate data, were applied to monthly simulated data throughout the 2015–2100 period. The ratio was derived for each climate variable separately. The CO_2_ and total CO_2_ equivalent GHG concentrations for the selected SSP scenarios needed to estimate future N deposition were obtained from [42].

Measurements of N deposition for Canadian forests during 1983–1994 made by the Canadian Air and Precipitation Monitoring Network [18] were used to produce a N deposition map for Canada, and then extrapolate it temporally based on the relationship between deposition rate and historical national greenhouse gas emissions [10]. Drainage, soil water characteristics, and soil texture (fractions of sand, silt, clay, and organic matter) were compiled from the Soil Landscape of Canada database [1]. Missing soil data values were interpolated based on a nearest neighbour scheme. The 30-arcsec digital elevation model data used to calculate the wetness index and local slopes for simulating the horizontal redistribution of soil water was derived from the Canadian Digital Elevation Data at 1:250,000 scale by the Canadian Forest Service [36].

The LAI, NPP, and wildfire fire disturbance data used in the study are described in detail by [26]. The LAI was derived from the moderate resolution imaging spectroradiometer (MODIS) 8-day reflectance observations and validated using field measurements collected in 2010 in 32 sample plots established in unmanaged forest in the southwestern part of the FNO. Reference NPP for 2004 was simulated using the BEPS model. Two sets of field measurements were used (1) to validate the BEPS reference NPP simulations and (2) to develop NPP-age curves used in simulations by InTEC model. Finally, data on historical fires for 1959–1999 were obtained from the Large Fire Database [63]. These data were supplemented with remote sensing observations to verify and expand the historical records to 2004. The historical fire data were used to assign forest age to the stands regenerating in the areas burned during 1959–2004 and to estimate the average annual wildfire rate that was used in simulations for 2005–2100. The rationale for choosing the historical wildfire rate in simulations of future C stocks is presented in "Discussion" section.

Carbon stocks for the FNO forests were simulated using historical data for 1901–2014 and using five SSP scenarios for 2015–2100. For each SSP scenario, two simulations were performed: with and without wildfires. The results for simulated C pools are combined in live vegetation, SOC, and total ecosystem pools and presented in the form of C density (amount of C per unit area). For cross-model comparison we used the following three models within the Coupled Model Intercomparison Project (CMIP5) project for which soil and vegetation C stocks were available in fully coupled model simulations [64]: the Canadian Earth System Model version 2 (CanESM2), the Hadley Centre Global Environmental Model version 2 (HadGEM2-ES) and the Institut Pierre Simon Laplace Climate Model 5A (IPSL-CM5A-LR). We used three experiments simulated by these models [64]: the ‘historical’ experiment for 1901 − 2005 is driven by prescribed CO_2_, aerosol, solar forcing, climate and land use change forcing; the ‘esmFdbk2’ experiment for 2006 − 2100 (referred to as “Climate”) with constant pre-industrial CO_2_ for physiological effects on vegetation and RCP4.5 greenhouse gas concentrations for radiative calculations; and the ‘esmFixClim2’ experiment for 2006 − 2100 (referred to as “CO_2_”) with RCP4.5 greenhouse gas concentrations for physiological effects on vegetation and pre-industrial greenhouse gas concentrations for radiative calculations. Data simulated for the above-listed scenarios were acquired for a geographic area spanning 50 − 55°N and 82 − 95°W, encompassing the entire forested area of the FNO.

## Supplementary Information

**Additional file 1.** Histotical and projected C stocks and fluxes.

## Data Availability

Results of the analysis and references to the data sources are included in the Additional file 1 accompanying this paper.

## Abbreviations

BEPS: Boreal Ecosystem Productivity Simulator
C: Carbon
CMIP: Coupled Model Intercomparison Project
DOM: Dead organic matter
ESM: Earth System Model
FMU: Forest management unit
FNO: Far North of Ontario
GHG: Greenhouse gas
LAI: Leaf area index
N: Nitrogen
NPP: Net primary productivity
SOC: Soil organic carbon
SSP: Shared socioeconomic pathway
